# Getting a Grip on piRNA Cluster Transcription

**DOI:** 10.1016/j.cell.2014.05.022

**Published:** 2014-06-05

**Authors:** Alexandra Sapetschnig, Eric A. Miska

**Affiliations:** 1 Wellcome Trust/Cancer Research Gurdon Institute, Department of Biochemistry and Department of Genetics, University of Cambridge, Tennis Court Road, Cambridge CB2 1QN, UK

## Abstract

The generation of piRNAs from long primary transcripts requires specialized factors that distinguish these precursors from canonical RNA polymerase II transcripts. Mohn et al. and Zhang et al. provide evidence that in *Drosophila melanogaster* noncanonical transcription coupled with splicing inhibition differentiates piRNA precursors from mRNAs and ensures their correct processing.

## Main Text

A significant proportion of eukaryotic genomes consists of transposable and other repetitive elements. PIWI interacting RNAs (piRNAs) are a germline-specific class of small noncoding RNAs that protect the genome of animal germ cells from the action of transposable elements, which—when deregulated—can cause DNA damage and sterility (Siomi et al., 2011). In *Drosophila melanogaster*, zebrafish, and mouse germ cells, primary sources of piRNAs are genomic clusters that usually consist of transposon fragments and remnants of other repetitive elements, representing the history of recent transposon activity in the genome of these organisms (Brennecke et al., 2007). These loci need to be expressed throughout germ cell development in order to maintain a functional pool of piRNAs. According to our current knowledge, most primary piRNAs are generated by the expression of long, single-stranded precursor RNAs, which are RNA polymerase II (Pol II) transcripts, followed by subsequent processing in specialized cytoplasmic foci (Li et al., 2013; Siomi et al., 2011). How piRNA cluster transcription is defined and how the precursors are distinguished from canonical Pol II transcripts in order to get processed correctly into mature piRNAs remained unclear. In this issue of *Cell*, Mohn et al. (2014) and Zhang et al. (2014) shed new light on the mechanisms underlying piRNA cluster expression in *D. melanogaster*.

*D. melanogaster* piRNA clusters can be mainly divided into two groups: uni-strand clusters that give rise to piRNAs that map predominantly to only one strand and dual-strand clusters where piRNAs originate from both DNA strands (Brennecke et al., 2007). Mohn et al. (2014) show here that uni-strand clusters exhibit hallmarks of canonical Pol II transcription such as a defined Pol II peak around the transcription start site (TSS), an enrichment of the active histone mark H3K4me2 at their putative promoters and the expression of 5′ methyl-guanosine-capped and terminated RNAs. This correlates with mouse piRNA cluster expression characteristics where piRNA production is also mostly restricted to one DNA strand (Li et al., 2013). How these uni-strand clusters in *D. melanogaster* and piRNA clusters in mice are distinguished from very similar looking canonical mRNA-producing loci and how those RNAs are funneled into the piRNA processing machinery still remains to be answered.

Dual-strand clusters in contrast seem to be unique in flies: they lack a clear promoter (as defined by the lack of H3K4me2 peaks), 5′ methyl-guanosine caps, and clear transcription termination and seem to rather rely on Pol II read-through transcription from convergent neighboring gene pairs or noncanonical transcription initiation (Mohn et al., 2014). In addition, transcripts do not undergo splicing as they retain sequences that show intron-like features (Zhang et al., 2014). This noncanonical transcription and the presence of uncapped transcripts would usually lead to transcription termination and decay of the RNA. However, these unusual transcripts from dual-strand clusters successfully escape this fate to be processed into mature piRNAs. How do they achieve this?

**Figure 1:**
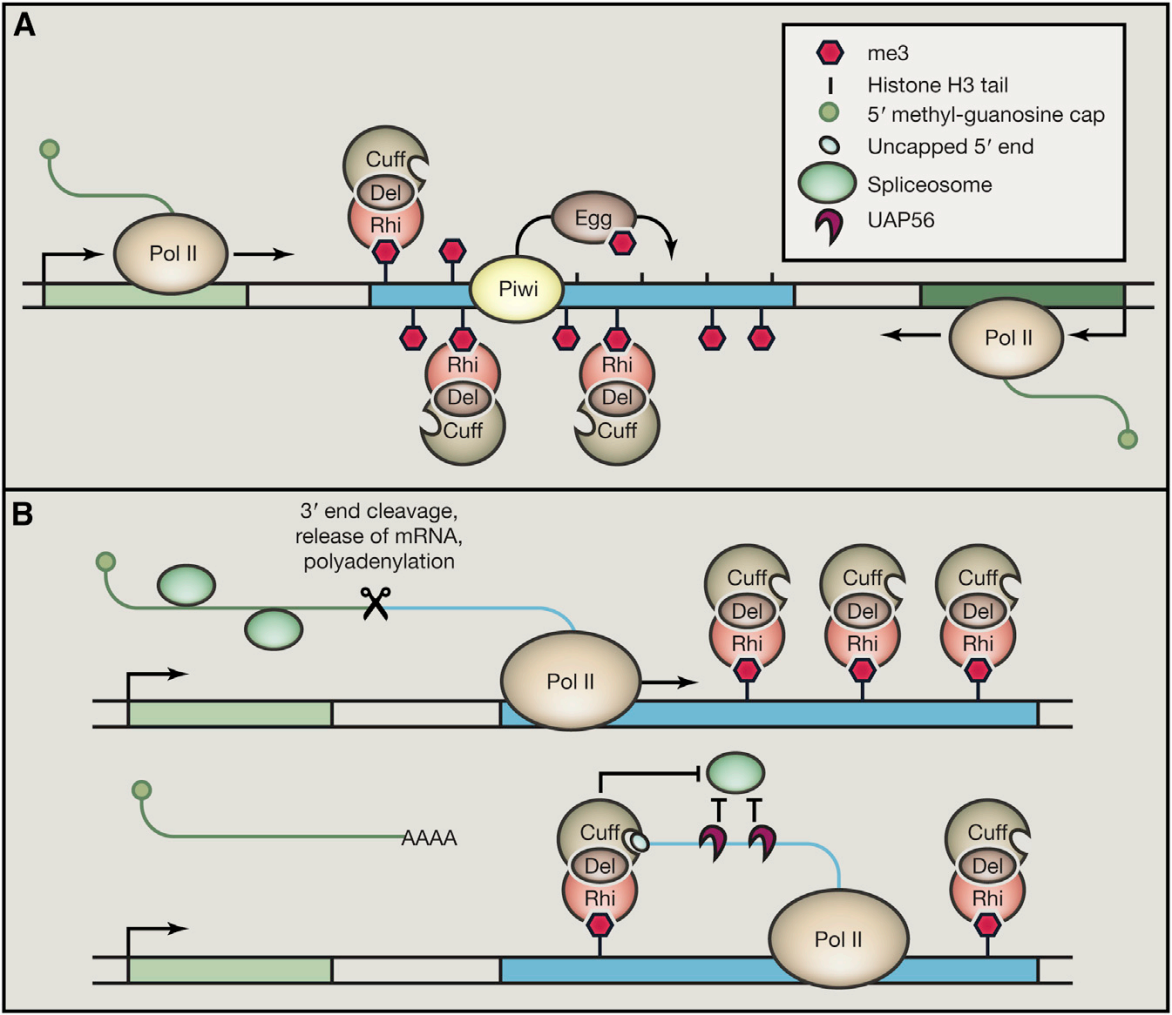
Model for Dual-Strand piRNA Cluster Expression in *Drosophila melanogaster* (A) Dual-strand cluster (blue box) transcription is achieved by read-through transcription from convergent neighboring genes (green boxes) or by noncanonical transcription inititation by RNA polymerase II (Pol II). piRNA-mediated recruitment of Piwi to some source loci leads to H3K9 trimethylation (me3) by Eggless (Egg) and subsequent Rhino (Rhi) recruitment in complex with Deadlock (Del) and Cuttoff (Cuff). This licenses dual-strand cluster expression. (B) Rhi binding to chromatin brings Cuff into close proximity to the newly formed 5′ end of a nascent piRNA precursor transcript after the upstream transcript has undergone 3′ end processing. Cuff binding prevents degradation of the transcript and probably inhibits splicing together with UAP56, which marks the precursor for export and processing in perinuclear bodies.

Mohn et al. (2014) and Zhang et al. (2014) now provide the evidence that specific factors, previously identified to be required for dual-strand cluster expression and the generation of dual-strand cluster-derived piRNAs, are crucial to maintain this noncanonical expression. The heterochromatin protein Rhino (Rhi) and the Rai1-like transcription termination cofactor Cutoff (Cuff) together with the protein Deadlock (Del) bind as a complex to dual-strand-cluster chromatin probably via the H3K9me3-binding activity of Rhi (Figure 1A). Specifically, Rhi seems to act as a licensing factor since its binding strongly correlates with cluster expression and piRNA production (Klattenhoff et al., 2009; Mohn et al., 2014; Zhang et al., 2014), and it seems to distinguish piRNA loci from other heterochromatic regions in the genome that carry H3K9me3 marks. Interestingly, binding of Rhi requires the Piwi protein at some loci (Mohn et al., 2014) and the H3K9 methyltransferase Eggless (Egg) (Rangan et al., 2011), suggesting that a feedforward loop for piRNA production and piRNA-induced heterochromatin formation exists. It remains to be seen how the piRNA-producing loci are mechanistically distinguished from transposon-expressing loci that get silenced by heterochromatin formation via Piwi-piRNA complexes. Rhi binding brings the putative termination cofactor Cuff in close proximity to the nascent piRNA precursor transcript, which—after 3′ end processing of the upstream protein-coding transcript—exhibits an incompletely capped 5′ end (Figure 1B). Despite being a homolog of 5′ to 3′ exonucleases required for termination of Pol II transcription (Jiao et al., 2013), putative binding of Cuff to the 5′ end of the piRNA precursors does not lead to termination of transcription and rather protects them from degradation presumably since it lacks critical amino acids of its functional homologs (Pane et al., 2011).

In addition, Rhi, Cuff, and the DEAD box helicase UAP56, which is also crucial for piRNA biogenesis from dual-strand clusters (Zhang et al., 2012), are all required to inhibit splicing of the precursor RNA. Inhibition of splicing might be a consequence of preventing the Cap-binding complex to bind, which, in turn, can recruit the spliceosome. Alternatively, UAP56 may directly inhibit splicing by an unknown mechanism (Figure 1B). In an elegant experiment with a germline integrated and expressed transgene, Zhang et al. (2014) showed that tethering of Rhi to an artificial target locus (presumably by in turn recruiting Cuff and UAP56) is sufficient to prevent splicing of a transcript. Altogether, these findings suggest that it is the combined action of Rhi, Cuff, and UAP56 that allows for read-through transcription and the expression of noncanonical transcripts.

However, expressing a transcript from a Rhi-bound uni-strand locus is not sufficient to produce piRNAs. De novo production of piRNAs can be only induced by tethering Rhi to both strands of complementary transgenes suggesting that the dual-strand nature of the piRNA clusters is a prerequisite for the expression of piRNAs (Zhang et al., 2014). The double-stranded RNA (dsRNA) processing RNases Dicer-1 and Dicer-2, which are required for microRNA and small interfering RNA (siRNA) generation, respectively, seem not to be required for piRNA biogenesis (Siomi et al., 2011). In contrast, the presence of complementary transcripts with the ability to from dsRNA could yield in the production of siRNAs from these loci by the action of Dicer-2. But those small RNAs are not prevalent from the locus. Is the formation of dsRNA prevented? If yes, it will be exciting to see what the molecular mechanism behind this could be, and how exactly the complementary nature of the dual-strand clusters contributes to the generation of piRNAs.
